# Risk Factors for Cervical Cancer in Ghana

**DOI:** 10.1002/cnr2.2124

**Published:** 2024-06-21

**Authors:** Yvonne Nartey, Kwabena Amo‐Antwi, Philip C. Hill, Edward T. Dassah, Richard H. Asmah, Kofi M. Nyarko, Ramatu Agambire, Thomas O. Konney, Joel Yarney, Nelson Damale, Brian Cox

**Affiliations:** ^1^ Department of Adult Health, School of Nursing and Midwifery University of Ghana Legon Ghana; ^2^ School of Medicine and Dentistry Kwame Nkrumah University of Science & Technology/Komfo Anokye Teaching Hospital Kumasi Ghana; ^3^ Centre for International Health, Department of Preventive and Social Medicine, Dunedin School of Medicine University of Otago Dunedin New Zealand; ^4^ Department of Biomedical Sciences University of Health & Allied Sciences, School of Basic and Biomedical Sciences Ho Ghana; ^5^ Disease Control and Prevention Department Ghana Health Service Accra Ghana; ^6^ Department of Nursing Garden City University College Kumasi Ghana; ^7^ National Centre for Radiotherapy and Nuclear Medicine Korle Bu Teaching Hospital Accra Ghana; ^8^ Department of Obstetrics and Gynaecology Korle Bu Teaching Hospital Accra Ghana; ^9^ Hugh Adam Cancer Epidemiology Unit, Department of Preventive and Social Medicine Dunedin School of Medicine, University of Otago Dunedin New Zealand

**Keywords:** case–control, cervical cancer, cofactors, Ghana, risk factors

## Abstract

**Background:**

The major burden of cervical cancer occurs in low‐ and middle‐income countries. In Ghana, it is the second most common cancer among women. Infection with high‐risk human papilloma virus (HPV) has been established as the cause of cervical cancer. As such, it is important to identify risk factors that may affect progression from HPV infection to cancer.

**Aims:**

We assessed the risk factors assocaited with cervical cancer in Ghana.

**Methods:**

To identify the risk factors for cervical cancer, we conducted an unmatched case–control study in two hospitals in Ghana where most cervical cancer cases are diagnosed. Women with histologically confirmed cervical cancer were the cases, whereas women without cancer seeking care at the two hospitals were controls. A structured questionnaire was administered to the women, after which cervical samples were sent for HPV deoxyribonucleic acid (DNA) testing.

**Results:**

Overall, 206 cases and 230 controls were recruited. After adjusting for possible confounders, women with the highest educational level had a significantly lower risk of cervical cancer than those with no or little formal education. Parity was a major risk factor (odd ratio [OR] for five or more children = 7.9; 95% CI: 2.3–27.6), with risk increasing with increasing parity (*p* for trend <0.001). Women reporting the use of a homemade sanitary towel during menstruation also had an increased risk of cervical cancer compared with women who used a pad (OR: 7.3; 95% CI: 2.5–22.0).

**Conclusion:**

In this Ghanaian population, high parity and poor personal hygienic conditions were the main contributing factors to the risk of cervical cancer after adjustment for the presence of high‐risk HPV genotypes.

**Abbreviations**
ADC: adenocarcinoma
CI: confidence interval
DNA: deoxyribonucleic acid
FIGO: International Federation of Gynaecology and Obstetrics
HPV: human papillomavirus
IUD: intrauterine device
KATH: Komfo Anokye Teaching Hospital
KBTH: Korle Bu Teaching Hospital
OCs: oral contraceptives
ORs: odds ratios
SCC: squamous cell carcinoma

## 1 Introduction

The incidence and mortality rates for cervical cancer in Sub‐Saharan Africa are among the highest in the world. Recent estimates indicate an age‐standardised incidence of 33.4 per 100 000 person‐years [1]. In West Africa, there were 31249 new cases of cervical cancer reported in 2022, with 511 in Togo, 2360 in Ivory Coast and 988 in Burkina Faso. In Ghana, an estimated 3072 women were diagnosed with the disease in 2022 and 1815 died from it [1].

Cervical screening outside of the private sector is very limited in Ghana, covering a low proportion of women. In England, the introduction of cervical screening in 1988 has had a significant impact in reducing the burden of the disease with screening available to women over 25 years old [2]. The high incidence and mortality rates of cervical cancer in Ghana are often attributed to late stage at diagnosis [3, 4, 5]. There is no evidence that treatment in the second‐line setting improves overall survival compared with best supportive care [6]. However, women in this situation are often symptomatic and relatively young. As such, treatment options that offer improvement in disease‐related symptoms, quality of life and prolongation of progression‐free survival are worthwhile [6].

Certain types of human papillomavirus (HPV) are currently recognised as the central etiologic factor of cervical cancer. There are limited studies on the prevalence of HPV among the Ghanaian population [7, 8, 9]. HPV prevalence has been reported to vary between 10.7% and 45.8% depending on the population studied [7, 9, 10, 11, 12, 13]. Although the majority of women infected with HPV clear the infection, in others, it persists and increases the risk of progression to invasive cervical cancer. Additionally, cervical cancer is infrequent in women under 25 years of age. This has led to the conclusion that other risk factors may contribute to the progression of HPV to cancer.

High parity, long‐term use of oral contraceptives (OCs) and smoking among others have been implicated in the progression of HPV infection to cancer [14, 15, 16, 17, 18, 19]. The importance of these factors are recognised in high‐income countries. Studies of their role, and that of other possible risk factors, in cervical cancer in low‐ and middle‐income countries, particularly in Africa, are needed. Specific cofactors, such as poor personal hygiene and polygamy, have been reported in some low‐ and middle‐income countries [18, 20]. Further research is needed to comprehensively understand their significance in various settings, including African countries. Currently, there is a lack of literature on cofactors associated with cervical cancer specifically in Ghana. Enhanced knowledge of factors influencing cervical cancer development is crucial for effective prevention strategies. This study aims to assess the contribution of various risk factors in the development of cervical cancer in Ghana.

## 2 Methods

### 2.1 Study Design and Setting

A hospital‐based case–control study control with 206 cases and 230 control women was conducted in two hospitals in Ghana: Korle Bu Teaching Hospital (KBTH) and Komfo Anokye Teaching Hospital (KATH). The method for this study has previously been published [7].

### 2.2 Study Population

Cases were defined as women residing in Ghana for at least 3 years with a histological diagnosis of invasive cervical cancer, who had not received previous treatment for the disease, and were aged 18–95 years of age. The control group comprised of women without cervical cancer or a history of cervical cancer who were attending the same hospital. Control women were excluded if they had diseases associated with known risk factors for cervical cancer, such as breast cancer, endometrial cancer, ovarian cancer, colon cancer or tobacco‐related diseases (examples: coronary heart disease, lung cancer or chronic bronchitis). Women who came to the hospital for family planning services were also excluded as controls.

### 2.3 Interview and Sample Collection

All consenting women were administered a standardised questionnaire by trained interviewers covering demographic characteristics, sexual behaviour, reproductive and contraceptive history, genital hygiene and screening history. An effort was made to keep the interviewers blinded to the case or control status of the study subjects. A gynaecological examination was then performed with the collection of biopsy specimens in the cases and exfoliated cells in the controls. The smears were sent to the cytology laboratory of the School of Biomedical and Allied Health Sciences, University of Ghana, Korle‐Bu, Accra. Exfoliated cervical cells left on the brush were recovered from the brush, after preparation of the cervical smears, by washing into a pre‐labelled tube containing DNAGuard (Biometrica, San Diego, USA), to preserve cellular DNA at room temperature until molecular analysis of HPV DNA and typing. The cytology results were classified using the Bethesda system [21] and pre‐cancer lesion were managed according to the World Health Organization protocol [22].

Approval for this study was granted by the University of Otago Ethics (Health) Committee, Ghana Health Service Ethical Committee (Protocol ID: GHS‐ERC: 01/05/14) and the Committee on Human Research, Publication and Ethics of the Kwame Nkrumah University of Science and Technology (KNUST) and KATH, Ghana (CHRPE/AP/661/19). Written informed consent was obtained from all women who participated in the study. They were informed that participation was voluntary, and they had the right to withdraw from the study at any time.

### 2.4 Statistical Analysis

The data were imported from Excel into STATA version 15.1 (STATA Corporation, College Station, TX). Descriptive statistics were calculated for patient socio‐demographic, tumour and lifestyle factors. Odds ratios (ORs) and 95% confidence intervals (CIs) were calculated as approximations of the relative risks by unconditional regression. The statistical significance of trends for ORs was assessed by considering the categorical variables as a continuous variable in the logistic analysis. A *p*‐value of less than 0.05 was considered statistically significant.

To examine the possibility of confounding in the data, the frequency of exposure of control subjects to the various risk factors according to potential confounding variables, adjusted for age at diagnosis (age at interview for controls), was estimated. Variables yielding an adjusted OR appreciably different from the unadjusted estimate were identified. There were five variables which produced changes in the estimate OR and had a *p*‐value of more than 0.05: age at diagnosis, age at first marriage, region of residence, ethnicity and the presence of high‐risk oncogenic HPV. These were included in the final analysis. Age at diagnosis was included as a continuous variable.

## 3 Results

Overall, 209 women diagnosed with cervical cancer met inclusion criteria for recruitment. Some women met inclusion criteria but were not recruited. Three women (1.4%) with cervical cancer who met inclusion criteria were too ill and died before they could be recruited. Interviews were completed for 206 (98.6%) eligible women.

Two‐hundred thirty‐eight control women were eligible for recruitment. Interviews were completed for 230 (96.6%) of these women. One woman (0.4%) declined to be interviewed. Three were not recruited as two were vomiting and one other had a major speech defect that made interview impractical. The main diagnostic categories of the control subjects included in the study were benign disorders of the genital tract and menstrual disorders.

### 3.1 Patient Characteristics

Nearly 122 (59.2%) women with cervical cancer were recruited at KBTH compared with 84 (40.8%) from KATH. Eighty‐five percent (85.4%) of women with cervical cancer were diagnosed with squamous cell carcinoma (SCC) (Table 1). International Federation of Gynaecology and Obstetrics (FIGO) stage III was the most common stage at presentation. For 16.5% of the women the cancer was unstaged. Moderately differentiated cervical cancer was the most common pathological grade observed (25.2%).

**TABLE 1:** Clinical characteristics of women with cervical cancer.

Characteristic	Number (206)	%
Histological type		
SCC	176	85.4
ADC	7	3.4
Adenosquamous	1	0.5
Other	6	2.9
Unknown	16	7.8
Grade of differentiation		
Well differentiated	18	8.7
Moderately differentiated	52	25.2
Poorly differentiated	30	14.6
Undifferentiated	40	19.4
Unknown	66	32.1
FIGO stage		
Stage I	32	15.5
Stage II	44	21.4
Stage III	82	39.8
Stage IV	14	6.8
Unstaged	34	16.5

Abbreviations: ADC: adenocarcinoma; FIGO: International Federation of Gynaecology and Obstetrics; SCC: squamous cell carcinoma.

There was a significant difference between cases and controls in terms of age at first marriage, age at menarche, number of pregnancies, parity and abortion, but not age at first intercourse or age at first birth (Table 2). A high number of pregnancies and high parity were more common in cases than controls (*p* < 0.001). There was no statistically significant difference between cases and controls who used OCs or intrauterine device (IUD). Furthermore, smoking was not significantly more prevalent in cases than controls (*p* = 0.58). Having a previous cervical smear was uncommon in both cases and controls (Table S1).

**TABLE 2:** Distribution of cervical cancer cases and controls by selected reproductive factors.

	Cases (*n* = 206)	Controls
Characteristic	*n* (%)	*n* (%)
Age at first marriage (years)		
≤16	13 (6.5)	6 (3.7)
17–18	29 (14.5)	7 (4.4)
19–20	61 (30.5)	25 (15.5)
21–22	25 (12.5)	15 (9.3)
≥23	72 (36)	108 (67.1)
Unknown	0	0
Age at first sexual intercourse (years)^a^		
≤15	16 (7.9)	28 (12.4)
16–17	40 (19.9)	39 (17.3)
18–19	60 (29.9)	65 (28.7)
20–21	50 (24.9)	52 (23)
≥22	35 (17.4)	42 (18.6)
Unknown	5	1
Age at menarche (years)		
≤12	4 (2)	28 (12.2)
13–14	23 (11.2)	62 (26.9)
≥15	178 (86.8)	140 (60.9)
Unknown	1	0
Number of pregnancies		
0–2	16 (7.8)	136 (59.1)
3–4	45 (21.8)	44 (19.1)
≥5	145 (70.4)	50 (21.8)
Parity		
0–2	37 (17.9)	186 (80.9)
3–4	63 (30.6)	32 (13.9)
≥5	106 (51.5)	12 (5.2)
Age at first birth (years)		
≤16	13 (6.6)	9 (7.4)
17–18	26 (13.3)	11 (9.1)
19–20	53 (27.1)	26 (21.5)
21–22	32 (16.3)	21 (17.4)
≥23	72 (36.7)	54 (44.6)
Unknown	6	1
OC use		
Never	160 (78.4)	174 (76.3)
Ever	44 (21.6)	54 (23.7)
Unknown	2	2

^a^ Excludes three women who had not had sexual intercourse.

### 3.2 Socio‐Demographic Factors and Risk of Cervical Cancer

Socio‐demographic factors and their association with the risk of cervical cancer are shown in Table 3. There was a statistically significant trend towards increasing odds with increasing age at diagnosis (*p*‐value for linear trend, <0.001). The risk of cervical cancer was higher among women who lived in semi‐urban regions compared with those in a metropolis (OR_c_: 26.3; 95% CI: 2.4–284.1) and was significant with adjustment for the presence of high‐risk oncogenic HPV. However, the small number of controls who lived in semi‐urban regions meant this estimate was imprecise.

**TABLE 3:** Socio‐demographic factors and risk of cervical cancer in Ghana.

Characteristic	Cases (*n* = 206)	Controls (*n* = 230)			
	*n* (%)	*n* (%)	OR_a_ (95% CI)	OR_b_ (95% CI)	OR_c_ (95% CI)
Age group (years)					
≤39	13 (6.3)	159 (69.1)	1	1	1
40–49	43 (20.9)	52 (22.6)	10.1 (5.1–20.3)	8.4 (3.6–19.5)	5.2 (1.7–16.5)
50–59	59 (28.6)	26 (3.5)	90.2 (35.6–228.6)	61.2 (21.0–178.6)	26.8 (6.1–117.4)
≥60	91 (44.2)	231 (4.8)	101.2 (43.5–235.1)	63.2 (24.0–166.3)	23.1 (6.5–81.5)
Test for trend (*p*‐value)			<0.001	<0.001	<0.001
Region of residence					
Metropolis	104 (50.5)	103 (88.2)	1	1	1
Urban	82 (39.8)	15 (10.9)	3.5 (1.8–6.7)	3.0 (1.5–6.0)	1.0 (0.3–3.0)
Semi‐urban	20 (9.7)	26 (0.9)	15.9 (3.2–80.6)	12.7 (2.4–68.6)	26.3 (2.4–284.1)
Ethnicity					
Ga/Adangbe	15 (7.3)	38 (16.5)	1	1	
Ewe	35 (17)	26 (11.3)	3.9 (1.3–11.6)	4.4 (1.3–15.2)	
Akan	121 (58.7)	132 (57.4)	2.3 (0.9–5.8)	2.2 (0.9–15.2)	
Other	35 (17)	34 (14.8)	5.9 (2.0–17.6)	3.7 (2.0–15.2)	
Marital status					
Single	3 (1.5)	11 (30.9)	1	1	1
Married	97 (47.1)	231 (56.9)	8.7 (2.4–31.8)	2.3 (0.5–12.2)	3.4 (0.2–50.5)
Other	106 (51.4)	28 (12.2)	8.1 (2.0–32.2)	2.5 (0.4–14.2)	1.4 (0.1–24.7)
Formal education					
Tertiary	12 (5.9)	16 (20.2)	1	1	1
None	89 (43.6)	23 (10.1)	3.8 (1.4–10.6)	2.5 (0.7–9.2)	7.3 (0.8–70.1)
Primary	41 (20.1)	28 (12.3)	3.9 (1.4–11.4)	2.4 (0.7–8.2)	10.2 (1.1–95.3)
Secondary	62 (30.4)	131 (57.4)	1.5 (0.6–3.8)	1.4 (0.5–4.1)	3.6 (0.5–27.9)
Unknown	2	2			

*Note:* OR_a_: adjusted for age at diagnosis; OR_b_: adjusted for age, age at first marriage, region of residence and ethnicity; OR_c_: adjusted for age at diagnosis, age at first marriage, region of residence, ethnicity and presence of high‐risk oncogenic HPV DNA.

Ewe and other ethnic groups compared with Ga/Adangbe had a higher risk of cervical cancer (OR_b_: 4.4; 95% CI: 1.3–15.2 for Ewe and OR_b_: 3.7; 95% CI: 1.0–13.5 for other). The OR for cervical cancer adjusted for age was significantly higher among women who were married compared with those who were single (OR_a_: 8.7; 95% CI: 2.4–31.8). However, the risk did not achieve statistical significance after adjustment for the presence of high‐risk oncogenic HPV in addition to other factors. The risk of cervical cancer was higher among women with only primary school education compared with those with tertiary education (OR_c_: 10.2; 95% CI: 1.1–95.3).

### 3.3 Socio‐Economic Factors and Risk of Cervical Cancer

Table 4 shows risk of cervical cancer by socio‐economic factors in Ghana. Women who used gas or an electric cooker were at lower risk of cervical cancer compared with those using firewood (OR_b_: 0.10; 95% CI: 0.03–0.27). The association persisted after adjustment for the presence of high‐risk oncogenic HPV (OR_c_: 0.03; 95% CI: 0.01–0.13). The use of charcoal was also associated with a lower risk of cervical cancer compared with firewood (OR_b_: 0.2; 95% CI: 0.1–0.5) and remained significant after adjustment for high‐risk oncogenic HPV (OR_c_: 0.12; 95% CI: 0.02–0.58). To further explore the risk associated with firewood usage, data on firewood usage in cooking per week were collected. However, responses were too few for statistical analysis. Reporting of poor kitchen ventilation was statistically associated with an increased risk of cervical cancer after adjustment for age (OR_a_: 5.4; 95% CI: 1.5–19.5), but did not reach statistical significance after adjustment for high‐risk oncogenic HPV and other factors (OR_c_: 2.4; 95% CI: 0.4–14.4). The presence of water (OR_b_: 0.5; 95% CI: 0.3–0.9) and toilet facilities (OR_b_: 0.3; 95% CI: 0.1–0.6) at home were all associated with a statistically significant reduced risk of cervical cancer after adjustment.

**TABLE 4:** Socio‐economic factors and risk of cervical cancer in Ghana.

Characteristic	Cases (*n* = 206)	Controls (*n* = 230)			
	*n* (%)	*n* (%)	OR_a_ (95% CI)	OR_b_ (95% CI)	OR_c_ (95% CI)
Method of cooking					
Firewood	79 (38.7)	8 (3.5)	1	1	1
Charcoal	74 (36.3)	64 (27.9)	0.1 (0.1–0.3)	0.2 (0.1–0.5)	0.12 (0.02–0.58)
Gas/electric	51 (25)	157 (68.6)	0.06 (0.02–0.16)	0.1 (0.03–0.27)	0.03 (0.01–0.13)
Unknown	2	1			
Condition of kitchen					
Good	190 (92.7)	223 (97.4)	1	1	1
Poor	15 (7.3)	6 (2.6)	5.4 (1.5–19.5)	3.5 (0.8–15.6)	2.4 (0.4–14.4)
Unknown	1	1			
Water facility					
No	120 (58.3)	76 (33.0)	1	1	1
Yes	86 (41.7)	154 (67.0)	0.4 (0.2–0.7)	0.5 (0.3–0.9)	0.2 (0.1–0.6)
Toilet facility					
No	82 (40.0)	53 (23.0)	1	1	1
Yes	123 (60.0)	177 (77.0)	0.3 (0.2–0.5)	0.3 (0.1–0.6)	0.1 (0.04–0.32)
Unknown	1	0			
Type of toilet					
Water closet	60 (48.8)	116 (66.7)	1	1	1
KVIP	25 (20.3)	23 (13.2)	2 (0.8–5.1)	1.9 (0.6–6.1)	1.8 (0.3–10.2)
Bucket/pit latrine	38 (30.9)	35 (20.1)	1.4 (0.6–3.2)	1.3 (0.5–3.6)	1.5 (0.4–6.0)
Unknown	0	3			
Health insurance					
No	18 (8.7)	37 (16.2)	1	1	1
Yes	188 (91.3)	191 (83.8)	1.2 (0.6–2.8)	1.3 (0.5–3.1)	1.3 (0.2–7.2)
Unknown	0	2			
Household size					
≤5	130 (64)	144 (63.4)	1	1	1
6–10	55 (27.1)	52 (22.9)	1.1 (0.6–2.0)	1.1 (0.5–2.2)	1.6 (0.6–4.7)
11–15	11 (5.4)	15 (6.6)	0.9 (0.3–2.9)	1.0 (0.2–3.9)	1.6 (0.1–19.9)
>16	7 (3.5)	16 (7.1)	0.5 (0.2–1.7)	0.2 (0.04–0.70)	0.3 (0.04–2.47)
Unknown	3	3			
Test for trend (*p*‐value)			0.451	0.063	0.696

*Note:* OR_a_: adjusted for age at diagnosis; OR_b_: adjusted for age at diagnosis, age at first marriage, region of residence and ethnicity; OR_c_: adjusted for age at diagnosis, age at first marriage, region of residence, ethnicity and presence of high‐risk oncogenic HPV DNA.

### 3.4 Hygienic Factors and Risk of Cervical Cancer

Table 5 presents the association between hygienic practices and risk of cervical cancer. No statistically significant association between lack of special care in cleaning the genitals and cervical cancer was found (OR_b_: 2.0; 95% CI: 0.9–4.4). The use of water to clean the genitalia was associated with an increased risk of cervical cancer in the subgroup for whom HPV DNA test results were available. The use of a homemade sanitary towel compared with commercial pads was associated with increased odds of developing cervical cancer, even in the subgroup with adjustment for high‐risk oncogenic HPV DNA status (OR_c_: 7.3; 95% CI: 2.5–22.0). When duration of use of homemade sanitary towels was explored, the risk increased with increasing duration of use (*p*‐value for linear trend, 0.025).

**TABLE 5:** Risk for cervical cancer by hygienic patterns in Ghana.

	Cases (*n* = 206)	Controls (*n* = 230)			
Characteristic	*n* (%)	*n* (%)	OR_a_ (95% CI)	OR_b_ (95% CI)	OR_c_ (95% CI)
Special care in washing genitals					
Ever	148 (73.3)	189 (82.9)	1	1	1
Never	54 (26.7)	39 (17.1)	1.5 (0.8–2.9)	0.02 (0.9–4.4)	0.5 (0.1–3.1)
Unknown	3	2			
Water in washing genital					
Ever	119 (58.9)	142 (63.1)	1	1	1
Never	83 (41.1)	83 (36.9)	1.1 (0.6–1.9)	1.3 (0.7–2.4)	0.13 (0.03–0.69)
Unknown	4	5			
Type of sanitary towel^a^					
Pad	48 (23.6)	196 (85.2)	1	1	1
Homemade	155 (76.4)	34 (14.8)	4.5 (2.5–8.1)	2.9 (1.5–5.6)	7.3 (2.5–22.0)
Unknown	2	0			
Duration of use of homemade (years)^a^					
≤19	2 (1.3)	9 (26.5)	1	1	1
20–25	16 (10.5)	5 (14.7)	10.2 (1.5–69.5)	8.2 (1.0–66.4)	12.9 (0.6–260.9)
26–30	36 (23.5)	8 (23.5)	10.6 (1.7–67.0)	11.0 (1.41–86.6)	9.4 (0.4–256.3)
>30	99 (64.7)	12 (35.3)	14.3 (2.2–93.1)	12.1 (1.6–91.8)	4.5 (2.2–87.1)
Unknown	4	0			
Test for trend			−0.023		−0.781
Wash genitals after intercourse					
Yes	49 (24.4)	67 (29.5)	1	1	1
No	66 (32.8)	47 (20.7)	1.1 (0.5–2.2)	1.3 (0.5–3.0)	2.7 (0.6–11.8)
Sometimes	86 (42.8)	113 (49.8)	0.8 (0.4–1.4)	0.8 (0.4–1.6)	2.3 (0.7–8.0)
Unknown	5	3			

*Note:* OR_a_: adjusted for age at diagnosis; OR_b_: adjusted for age at diagnosis, age at first marriage, region of residence and ethnicity; OR_c_: adjusted for age at diagnosis, age at first marriage, region of residence, ethnicity and presence of high‐risk oncogenic HPV DNA.

^a^ One woman with cervical cancer used toilet roll.

## 4 Discussion

The current study identified both known and specific risk factors for the development of cervical cancer in Ghana including age, region of residence, ethnicity, use of homemade sanitary towel, method of cooking and the availability of toilet and water facilities at home. It is the first case–control study of risk factors for cervical cancer in Ghana and one of only a few in the West African sub‐region.

We found that living in a semi‐urban region was associated with an increased risk of cervical cancer. This has been reported elsewhere [23, 24, 25]. The association persisted after adjustment for the presence of high‐risk oncogenic HPV. Possible explanations for the association between region of residence and risk of cervical cancer may include access to cervical screening. Alternatively, differences in the prevalence of cofactors that may enhance the progression of HPV positivity to cancer may contribute to the association. Regions in Ghana vary in terms of number of pregnancies, parity, use of OC and literacy level. Parity is higher in urban and semi‐urban regions compared with metropolis [26]. Conversely, the use of OC is prevalent in metropolitan regions [27, 28]. These factors may promote persistence of HPV infection which increases progression to cancer. There is increasing interest in the role of immunotherapy in advanced cervical cancer, as the causative role of HPV is well‐established [29]. A number of immunotherapy trials have been undertaken evaluating vaccine‐based therapies, adoptive T‐cell therapy and immune‐modulating agents in patients with advanced cervical [29, 30]. In Ghana, cervical screening centres were mostly concentrated in Accra and Kumasi, leaving the vast majority of people with no access to secondary prevention. However, screening uptake was low in this study indicating that this is not a likely explanation for the increased risk associated with semi‐urban residence. Whether the relationship is due to residual confounding or other factors is unclear.

There was an association between ethnicity and the risk of cervical cancer. This was also found in the descriptive study [3, 31]. Various other studies have reported an increased risk of cervical cancer among different ethnic groups [32, 33, 34, 35]. There are no published studies that have assessed the prevalence of risk factors for cervical cancer by ethnicity in Ghana. Some studies conducted elsewhere have reported an association between ethnicity and HPV DNA positivity [36, 37]. However, an analysis for an association between ethnicity and risk for cervical cancer with adjustment for the presence of high‐risk oncogenic HPV in addition to other factors could not be conducted due to the small numbers of subjects in this study. It is unclear what may have accounted for this association, but it may have been differences in prevalence of risk factors across ethnic groups.

The use of homemade sanitary towels was associated with an increased risk of cervical cancer in this study (OR: 7.3; 95% CI: 2.5–22.0). The risk also increased with the duration of use (*p* for linear trend was 0.055). This observation is in agreement with previous studies that have reported an association between the use of homemade sanitary towels and cervical cancer [18, 20]. Whether this association is due to incomplete adjustment for confounding or by a direct mechanism of action is unknown. However, concerns have been raised previously that insertion of potentially infected implements might transmit HPV [38]. However, the sanitary towels are likely to be for one women and not shared. The use of a homemade sanitary towel may not be associated with the acquisition of HPV DNA but rather its persistence. Re‐use of homemade sanitary towels may increase the risk of persistence of HPV DNA through unhygienic practices. This suggests that users of homemade sanitary towels may be more likely to have persistent presence of HPV DNA. In warm countries, it might be possible for viral particles in shed epithelial cells from genital warts, cervical fluids or in blood to be infective for sufficient time to be transmissible as fomites.

We found that individuals classified as ‘other ethnic groups’ in Ghana showed a heightened risk of cervical cancer. This suggests a potential association between ethnicity and cervical cancer incidence. However, our study's sample size may have been insufficient to accurately detect this association. Additionally, factors such as access to toilets, availability of water and the use of gas or electric stoves for cooking were correlated with a reduced risk of cervical cancer. Similar findings have been reported in other contexts [18, 20]. Nonetheless, these factors are likely indicators of socioeconomic status, which itself has been linked to cervical cancer risk.

The recruitment of control women through the Department of Obstetrics and Gynaecology (only at the gynaecology unit of the department) may have introduced bias in the results. This is a common problem in hospital‐based case–control studies. Women with gynaecological issues are more likely to have diseases that may predispose them to HPV DNA positivity or other cofactors for cervical cancer. Use of all women attending the hospital as the sampling frame for controls was not found to be feasible in the two hospitals as it was difficult for a gynaecologist to leave the Department of Obstetrics and Gynaecology to obtain a cervical smear from women in other hospital departments. It was also not feasible for women to move from one hospital department to the other for a smear as most departments were in a separate building. The approach might have been possible if cervical smears were not required. With 206 cases and 230 controls, the study had limited power to detect some of the factors associated with cervical cancer, especially for subgroup analysis. For example, due to the paucity of users, analysis could not be performed for duration of use of OC or IUD. Additionally, analyses were not able to be performed for some variables with adjustment for the presence or absence of high‐risk oncogenic HPV DNA. To reduce the risk of information bias, both study subjects and research assistants were blinded to the case–control status of the subjects. HPV DNA detection assays vary in their sensitivity and ability to detect multiple HPV types and may affect the proportion of HPV detected in this study. There is also potential for higher detection of HPV DNA among women with cervical cancer compared with controls might be due to the differences in the type of specimens used for HPV DNA ascertainment.

Ghana does not have a national cervical screening program. Most screening that occurs is opportunistic and in private healthcare settings. Knowledge on screening HPV and other risk factors associated with the development of cervical cancer is also limited [39, 40, 41]. Due to the late stage of diagnosis of most cervical cancer in Ghana, the majority of women will require some form of radiotherapy often with chemotherapy. However, the availability of radiotherapy is very limited in Ghana. There are only two national radiotherapy centres: Korle Bu Teaching Hospital, Accra and Komfo Anokye Teaching Hospital, Kumasi. The Swedish Ghana Medical Center in Accra offers radiotherapy and chemotherapy, but many patients cannot afford private healthcare. While the government subsidises the cost of treatment, transportation expenses hinder access for some women. Significant investments by international organisations and the Government of Ghana have been made in promoting visual inspection with acetic acid (VIA) through demonstration projects [42]. Although VIA was introduced in pilot studies between 2001 and 2005, attempts to scale up nationwide coverage have been unsuccessful due to various factors, including government commitment issues and competing health priorities [42]. Recently, the Catholic hospital, Battor at the North Tongu district began screening using HPV testing to screen women and have made significant progress [43].

## 5 Conclusion

The findings of this study are in keeping with established risk factors and cofactors reported elsewhere were also found in this study. It is clear that the relationship observed between various socioeconomic factors may be mediated by health seeking behaviour and access to cervical cancer preventive interventions. Knowledge on cervical cancer among women in Ghana is limited. Government intervention in reducing poverty may help reduce the burden associated with cervical cancer. Certain groups may need targeting for education and increasing awareness on risk factors and preventive interventions.

## Author Contributions

**Conceptualization:** Yvonne Nartey, Brian Cox and Philip C. Hill. **Data curation:** Yvonne Nartey, Brian Cox, Philip C. Hill and Kwabena Amo‐Antwi. **Formal analysis:** Yvonne Nartey, Brian Cox, Philip C. Hill and Kwabena Amo‐Antwi. **Methodology:** All authors. **Project administration:** Yvonne Nartey, Brian Cox, Philip C. Hill and Kwabena Amo‐Antwi. **Writing‐original draft:** Yvonne Nartey. **Writing‐review and editing**: All authors.

## Conflicts of Interest

The authors declare no conflicts of interest.

## Supporting information


Table S1.

## Data Availability

Data cannot be shared publicly because of ethical concerns. Data will be available from the Ghana Health Service.
